# The Effects of Stochastic Galvanic Vestibular Stimulation on Body Sway and Muscle Activity

**DOI:** 10.3389/fnhum.2020.591671

**Published:** 2020-12-14

**Authors:** Akiyoshi Matsugi, Kosuke Oku, Nobuhiko Mori

**Affiliations:** ^1^Faculty of Rehabilitation, Shijonawate Gakuen University, Daitou, Japan; ^2^Department of Rehabilitation, Kawasaki University of Medical Welfare, Kurashiki, Japan; ^3^Department of Neuromodulation and Neurosurgery, Graduate School of Medicine, Osaka University, Osaka, Japan; ^4^Department of Neurosurgery, Osaka University Graduate School of Medicine, Osaka, Japan

**Keywords:** stochastic resonance, noise stimulation, galvanic vestibular stimulation (GVS), somatosensory, body sway, muscle activity

## Abstract

**Objective:** This study aimed to investigate whether galvanic vestibular stimulation with stochastic noise (nGVS) modulates the body sway and muscle activity of the lower limbs, depending on visual and somatosensory information from the foot using rubber-foam.

**Methods:** Seventeen healthy young adults participated in the study. Each subject maintained an upright standing position on a force plate with/without rubber-foam, with their eyes open/closed, to measure the position of their foot center of pressure. Thirty minutes after baseline measurements under four possible conditions (eyes open/closed with/without rubber-foam) performed without nGVS (intensity: 1 mA, duration: 40 s), the stimulation trials (sham-nGVS/real-nGVS) were conducted under the same conditions in random order, which were then repeated a week or more later. The total center of pressure (COP) path length movement (COP-TL) and COP movement velocity in the mediolateral (Vel-ML) and anteroposterior (Vel-AP) directions were recorded for 30 s during nGVS. Furthermore, electromyography activity of the right tibial anterior muscle and soleus muscle was recorded for the same time and analyzed.

**Results:** Three-way analysis of variance and *post-hoc* multiple comparison revealed a significant increment in COP-related parameters by nGVS, and a significant increment in soleus muscle activity on rubber. There was no significant effect of eye condition on any parameter.

**Conclusions:** During nGVS (1 mA), body sway and muscle activity in the lower limb may be increased depending not on the visual condition, but on the foot somatosensory condition.

## Introduction

The vestibular complex system is important for postural control (Dunlap et al., 2019). Head movement accompanies body sway and activates the vestibular nerve and contraction of muscles for postural control, and disturbance of this vestibulospinal response is known to cause falls (Whitney et al., 2015). Intervention to modulate the gain of vestibulospinal response is important in physical rehabilitation (Whitney et al., 2015; Lawson et al., 2016). Recently, galvanic vestibular stimulation (GVS) (Fitzpatrick and Day, 2004) has been used to test vestibular function (Matsugi et al., 2017) and treatment options (Dlugaiczyk et al., 2019). Furthermore, it has been reported that stochastic electrical stimulation of the vestibular nerve improves the postural balance in young people (Inukai et al., 2018b), elderly people (Fujimoto et al., 2016; Inukai et al., 2018a) and patients with vestibular disorders (Fujimoto et al., 2018), with long aftereffects. Noise GSV (nGVS) (Wuehr et al., 2017) can modulate the threshold of motor responses from vestibular inputs (Wuehr et al., 2017) in a posture-dependent manner (Matsugi et al., 2020), resulting in improvements in balance (Fujimoto et al., 2016, 2018; Inukai et al., 2018b). One possible rationale for changes in the motor threshold is stochastic resonance, in which the addition of low-intensity noise affects the nonlinear systems, which induces a response against signals that are buried in natural noise within neural systems (Mcdonnell and Ward, 2011). In other words, the threshold of the vestibulospinal response is decreased during nGVS based on the stochastic resonance mechanism, resulting in a large body sway that may be inhibited.

There is currently insufficient evidence to ascertain ideal conditions for the proper enhancement of nGVS's stochastic resonance effects on postural stability. Vestibular, visual, and somatosensory information are important for postural control in humans (Horak et al., 1994); however, the effects of these on postural sway during nGVS are unknown. The body sway induced by square wave pulse GVS when eyes are closed is dramatically reduced upon opening the eyes (Fitzpatrick and Day, 2004), because the vestibulospinal reflex activated by square wave pulse GVS is affected by eye condition (Matsugi et al., 2017). Furthermore, the rubber foam, which manipulates somatosensory information from the foot and makes it unreliable, also has mechanical consequences on balance control and increases the effect of square wave pulse GVS on body sway (Fitzpatrick et al., 1994). Based on the findings of the square wave pulse GVS effect in eye and foot conditions, the effect of nGVS on postural sway may also be affected by visual and somatosensory inputs. Therefore, this study aimed to investigate the effects of visual (eyes open/closed) and somatosensory inputs (from the feet using a hard platform/a platform with rubber foam) on postural sway.

Square-wave pulse GVS induces muscle contractions (Fitzpatrick et al., 1994; Ali et al., 2003) and modulates spinal motoneuron pool excitability (Matsugi et al., 2017, 2020; Okada et al., 2018). Therefore, nGVS may increase muscle activity for postural control. If there is a decrease in postural stability due to muscle contraction during nGVS, electromyography (EMG) in applicable muscle activity increases. However, there is currently insufficient evidence regarding the effect of nGVS on muscle activity, and the relationship between them. Therefore, we investigated muscle activity via EMG of the tibialis anterior (TA) and soleus (SOL) muscles during all examinations and analyzed how the activity was affected by visual signals and somatosensory signals from the foot.

## Methods

### Participants

Before the experiments, the appropriate sample size was estimated by analysis using G*power software (Version 3.1.9.4) (Faul et al., 2007) for analysis of variance (ANOVA) in repeated measure and within-between interaction. The type of power analysis was set to “A priori: Compute required sample size- given alpha, power, and effect size.” The effect size f was set to 0.5 (middle level), alpha error probability was set to 0.05, Power (1—beta error probability) was set to 0.95, correlation among repetitive measures was set to 0.5, and nonsphericity correction epsilon was set to 1. Then, the calculated sample size was 12. Therefore, we recruited 17 healthy subjects.

Seventeen healthy adults (mean age, 21.9 ± 4.6 years; 10 men and 7 women) participated in this study. None of the participants had a history of epilepsy or other neurological diseases. The ethics committee of Shijonawate Gakuen University approved the experimental procedures (approval code: 29–4), and this study was conducted according to the principles and guidelines of the Declaration of Helsinki with the understanding and written consent of each participant.

### General Procedure

This study was conducted in a sham-controlled, crossover, double-blind design. The assessors that recorded the center of pressure (COP) of the participants' feet and performed electromyography (EMG) as well as participants, were blinded to the real- or sham-nGVS conditions.

All subjects participated in two examinations—the real-nGVS trial and sham-nGVS trial—at random, with an inter-test interval of more than 1 week. Baseline measurements without nGVS were conducted for 30 min before the nGVS condition was applied in both real and sham trials, meaning baseline measurements were conducted both days. Further, before baseline measurements were acquired, we confirmed that a 3 mA square wave pulse (GVS) (Okada et al., 2018), administered for 200 ms (Matsugi et al., 2017), prompted the body to sway to the anodal side when participants stood with their eyes closed, feet together, and head facing forward (Fitzpatrick and Day, 2004; Matsugi et al., 2017). This was done to test whether they were responders to GVS.

### Foot Center of Pressure Measurements

**Figure 1:**
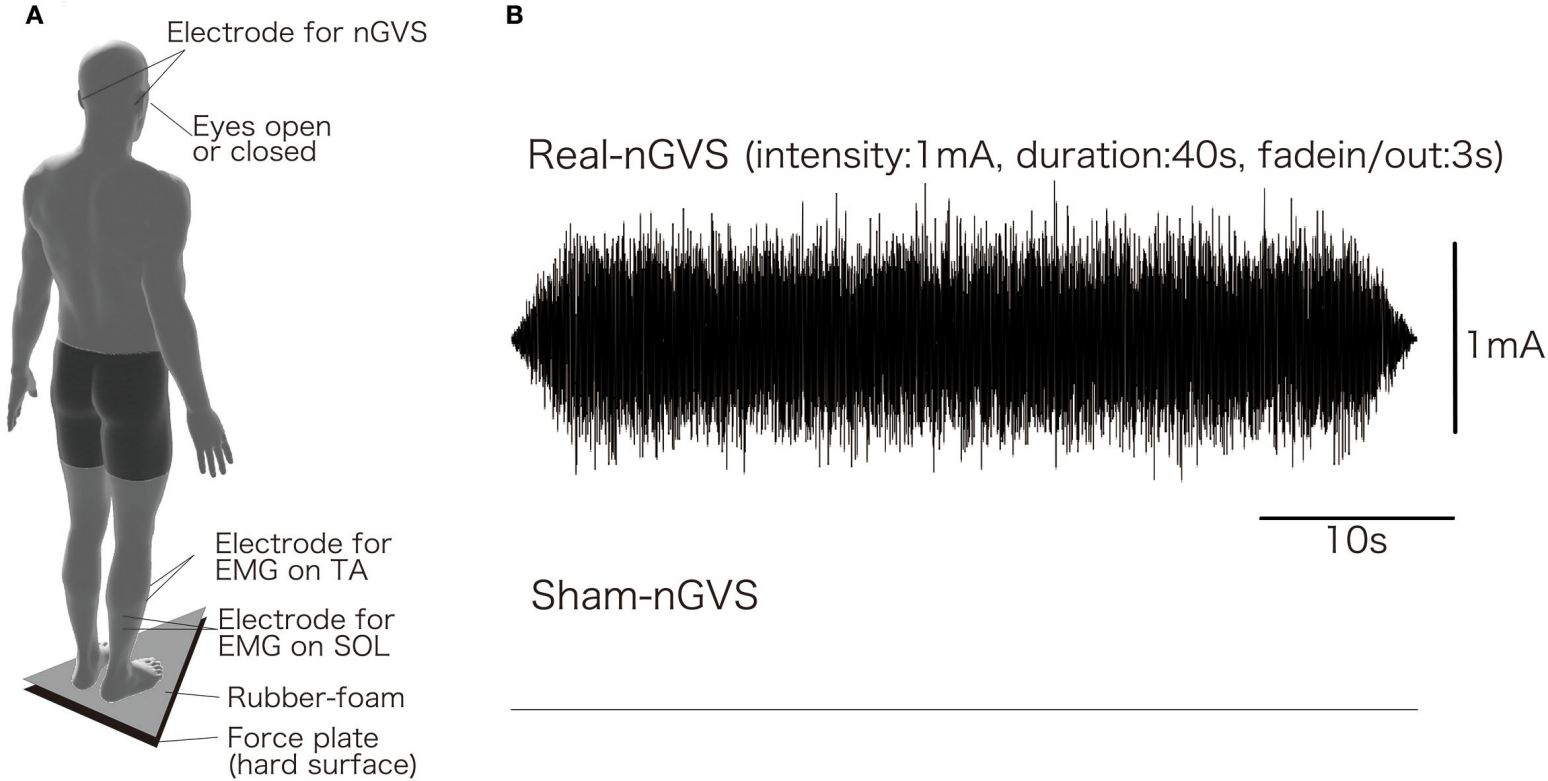
experimental setup **(A)** and typical wave-form of nGVS **(B)**.

Figure 1A shows the setup used to acquire the measurements. Subjects were asked to maintain an upright standing position on the pre-printed footprint, with both toes of the subjects outward at an angle of 15°, and with heel contact. The subjects were asked to look straight ahead with their head erect during each measurement and keep both upper limbs relaxed and lowered to the side. Furthermore, only during eyes open conditions, the subjects were asked to gaze at the target (a red magnet, 1 cm in diameter, attached to a whiteboard) 2 m away from the subject.

To estimate postural sway, the position of the center of pressure (COP) of each participant's foot while standing was measured at a sampling rate of 20 Hz. The position of the COP was calculated from the ground reaction force recorded using a force plate (Gravicorder G5500; Anima, Japan), and the total length (COP-TL) and velocity to the mediolateral (Vel-ML) and anterior-posterior (Vel-AP) directions were calculated in the same way as in our previous study (Matsugi et al., 2017). The measurements were conducted under the following conditions: eyes open or closed, using either rubber foam or rubber at all. Subjects were asked to maintain an upright standing position for 50 s for the following conditions, in a random order: (1) eyes open without rubber, (2) eyes closed without rubber, (3) eyes open with rubber foam, and (4) eyes closed with rubber foam. We used a rubber foam (Anima; thickness, 3.5 cm; tension strength, 2.1 kg/cm^2^) placed on the force plate, and in the hard platform condition, we removed this rubber.

### EMG Recording

Figure 1A shows the setup for EMG recording, which was performed in the same way as in our previous study (Matsugi et al., 2017). To record the EMG signals, two Ag/AgCl surface-recording electrodes were placed 2 cm apart on the right tibial anterior muscle (TA) and soleus muscle (SOL). The EMG signals were amplified via an amplifier (MEG-1200, Nihon Kohden, Japan) with a pass-band filter of 15 to 3 kHz. The EMG signals were converted to digital signals at a sampling rate of 10 kHz using an A/D converter (PowerLab 800S; AD Instruments, Colorado Springs, CO, USA), and the digital signals were stored on a personal computer.

### nGVS

Figure 1A shows the stimulation setup, and Figure 1B shows the typical waveform of nGVS. nGVS was conducted in almost the same way as in a previous study (Inukai et al., 2018b; Matsugi et al., 2020). nGVS was delivered via Ag/AgCl surface electrodes affixed to the right and left mastoid processes. DC-STIMULATOR PLUS (Eldith, NeuroConn GmbH, Ilmenau, Germany) was used to deliver random noise galvanic stimulation to the primary vestibular nerve. For nGVS in the “noise” stimulation mode, a random level of current was generated for every sample (sample rate 1,280 samples/s) (Moliadze et al., 2012; Inukai et al., 2018b; Matsugi et al., 2020). The intensity was set to 1 mA in this mode. Statistically, random numbers are normally distributed over time. The probability density follows a Gaussian bell curve, and all coefficients have a similar size in the frequency spectrum in this mode. A waveform was applied with 99% of the values located between−0.5 and + 0.5 mA, and only 1% of the current level was within the range of ±0.51 mA in this stimulation mode. The stimulation time was set to 40 s, and the current was ramped up and down before and after 3 s of stimulation. For the sham stimulation, direct current stimulation was performed, with the intensity set to 0 mA (sham-nGVS).

### Analysis

We calculated the COP-TL and average velocity of the COP movement in the mediolateral (Vel-ML) and anterior-posterior (Vel-AP) directions from the COP position. These were measured for 30 s in the baseline and stimulation trials. All EMG traces were rectified, and the average EMG amplitude was calculated. These parameters, which were COP-TL, Vel-ML, Vel-AP, EMG-TA, and EMG-SOL, were compared as a ratio to the baseline values (parameters/baseline values). To estimate the effects of the stimulation (sham and real) as well as from the visual (eyes open and closed), and somatosensory information from the foot (with and without rubber foam), we conducted a three-way analysis of variance (TW-ANOVA) with repeated measures. To estimate the effect size, the eta values were calculated. When an interaction effect was observed between the means of the parameter, an analysis of the simple main effect and Tukey's multiple comparison were conducted. Further, to estimate the correlation between changes in body sway and muscle activity, Pearson's correlation analysis was performed for all parameters. Statistical analyses were conducted using JASP software (version 0.9.2; University of Amsterdam, Amsterdam, the Netherlands) (Team, 2019). The alpha level was set at 0.05.

## Results

All subjects had body-sway to the anodal side during square wave pulse GVS before examination, indicating that all subjects were responders to GVS. None of the subjects showed any side effects, such as pain, dizziness, or discomfort, in any of the trials.

Table 1 shows the results of the test/baseline ratio in COP-TL, Vel-ML, Vel-AP, EMG-TA, and EMG-SOL. Table 2 shows the results from the TW-ANOVA, revealing that there were significant main effects of rubber in the EMG-SOL (*F* = 6.499, *p* = 0.0012, η^2^ = 0.046) and tat from stimulation in the Vel-AP (*F* = 6.559, *p* = 0.012, η^2^ = 0.047). There was a significant interactive effect of stimulation + rubber in the COP-TL (*F* = 6.252, *p* = 0.0014, η^2^ = 0.045), Vel-ML (*F* = 7.298, *p* = 0.008, η^2^ = 0.052), and EMG-SOL (*F* = 4.966, *p* = 0.0028, η^2^ = 0.046).

**Table 1:** Result of test/baseline ratio in COP-TL, Vel-ML, Vel-AP, EMG-TA, EMG-SOL.

**Stimulation**	**Eye**	**Rubber**	**COP-TL**	**Vel-ML**	**Vel-AP**	**EMG-TA**	**EMG-SOL**
Sham	Open	Off	1 (0.202)	1.062 (0.272)	0.957 (0.195)	0.998 (0.341)	0.984 (0.19)
		On	0.926 (0.135)	0.936 (0.183)	0.917 (0.113)	1.014 (0.645)	0.987 (0.284)
	Close	Off	0.939 (0.1)	0.957 (0.111)	0.929 (0.133)	0.843 (0.338)	0.956 (0.242)
		On	0.861 (0.176)	0.88 (0.217)	0.863 (0.22)	1.008 (0.413)	0.914 (0.185)
Real	Open	Off	0.928 (0.125)	0.906 (0.119)	0.964 (0.182)	0.953 (0.27)	1.028 (0.223)
		On	1.035 (0.211)	1.062 (0.267)	1.056 (0.253)	0.926 (0.39)	0.848 (0.265)
	Close	Off	0.965 (0.176)	0.983 (0.269)	1 (0.173)	0.997 (0.383)	1.049 (0.139)
		On	1.027 (0.295)	1.064 (0.355)	0.987 (0.236)	0.888 (0.336)	0.877 (0.224)

*The numbers indicate mean of test/baseline ratio (standard deviation). COP-TL, total path length of movement of foot center of pressure; Vel-ML, velocity of the COP movement in the mediolateral direction; Vel-AP, velocity of the COP movement in the anteroposterior direction; EMG-TA, averaged amplitude of the electromyography of the tibial anterior muscle*.

**Table 2:** Results from three-way ANOVA.

	**COP-TL**				**Vel-ML**				**Vel-AP**				**EMG-TA**				**EMG-SOL**			
	**Mean Square**	**F**	***p***	**η^2^**	**Mean Square**	**F**	***p***	**η^2^**	**Mean Square**	**F**	***p***	**η^2^**	**Mean Square**	**F**	***p***	**η^2^**	**Mean Square**	**F**	***p***	**η^2^**
Stimulation	0.111	3.198	0.076	0.023	0.068	1.205	0.274	0.009	0.246	6.559	**0.012**	0.047	0.021	0.127	0.722	9.701e−4	0.003	0.065	0.8	4.620e−4
Eye	0.02	0.575	0.449	0.004	0.014	0.25	0.618	0.002	0.028	0.759	0.385	0.005	0.051	0.312	0.577	0.002	0.006	0.112	0.738	8.034e−4
Rubber	5.869e−4	0.017	0.897	1.207e−4	0.002	0.04	0.843	2.832e−4	0.001	0.038	0.846	2.726e−4	0.004	0.024	0.876	1.866e−4	0.324	6.499	**0.012**	0.046
Stimulation ^*^ eye	0.052	1.493	0.224	0.011	0.121	2.155	0.145	0.015	0.005	0.128	0.721	9.208e−4	0.059	0.365	0.547	0.003	0.049	0.974	0.325	0.007
Stimulation ^*^ rubber	0.217	6.252	**0.014**	0.045	0.411	7.298	**0.008**	0.052	0.072	1.92	0.168	0.014	0.212	1.304	0.256	0.01	0.209	4.183	**0.043**	0.03
Eye ^*^ rubber	0.005	0.149	0.701	0.001	0.001	0.025	0.874	1.796e−4	0.037	0.985	0.323	0.007	0.01	0.06	0.806	4.611e−4	0.003	0.06	0.807	4.285e−4
Stimulation ^*^ eye ^*^ rubber	0.004	0.107	0.745	7.621e−4	0.033	0.592	0.443	0.004	0.013	0.349	0.556	0.003	0.113	0.695	0.406	0.005	0.006	0.122	0.728	8.706e−4
Residual	0.035				0.056				0.037				0.163				0.05			

*COP-TL, total path length of movement of foot center of pressure; Vel-ML, velocity of the COP movement in the mediolateral direction; Vel-AP, velocity of the COP movement in the anteroposterior direction; EMG-TA, averaged amplitude of the electromyography of the tibial anterior muscle; EMG-SOL, averaged amplitude of the electromyography of the soleus muscle; STIMULATION, real-nGVS/sham-nGVS; EYE, eyes-closed/eyes-open; RUBBER, with/without rubber foam; bold letters indicate statistically significant results (p < 0.05)*.

Because there was a significant stimulation in Vel-AP and with rubber in EMG-SOL, a *post hoc* comparison was done in Vel-AP to estimate the effect of stimulation and in EMG-SOL to estimate the effect of rubber. In Vel-AP, there was a significant difference between the real and sham (mean difference = 0.085, *t* = 2.561, *p* = 0.012), indicating a significant increment in the velocity of COP movement in the AP direction by real-nGVS. In EMG-SOL, there was a significant difference between rubber on and off (mean difference = 0.098, *t* = 2.549, *p* = 0.012), indicating an increment of EMG activity of SOL muscle by standing on rubber foam during nGVS.

Given that there was a significant interaction effect between stimulation and rubber, a *post hoc* comparison (Tukey's test) was performed in COP-TL, Vel-ML, and EMG-SOL (Table 3). There was significant difference between Real-nGVS vs. Sham-nGVS on rubber foam in COP-TL (mean difference = 0.137, *t* = 3.033, *p* = 0.015) and Vel-ML (mean difference=0.1555, *t* = 2.687, *p* = 0.04), meaning significant increment of COP-TL and Vel-ML by real-nGVS on the rubber foam condition. Further, there was a significant difference between rubber on vs. off in the Real-nGVS condition in EMG-SOL (mean difference=0.176, *t* = 3.249, *p* = 0.008), meaning a significant increment of EMG activity of SOL by standing on rubber foam in the real-nGVS condition.

**Table 3:** *Post-hoc* comparison.

		**COP-TL**				**Vel-ML**				**EMG-SOL**			
**Factor1**	**Factor2**	**Mean difference**	**SE**	***T***	***p*_**tukey**_**	**Mean difference**	**SE**	***t***	***p*_**tukey**_**	**Mean difference**	**SE**	***t***	***p*_**tukey**_**
Real, Roff	Sham, Roff	−0.023	0.045	−0.504	0.958	−0.065	0.058	−1.134	0.669	0.069	0.054	1.266	0.586
Real, Roff	Real, Ron	−0.084	0.045	−1.86	0.251	−0.118	0.058	−2.051	0.175	0.176	0.054	3.249	**0.008**
Real, Roff	Sham, Ron	0.053	0.045	1.173	0.645	0.037	0.058	0.636	0.92	0.088	0.054	1.623	0.37
Sham, Roff	Real, Ron	−0.061	0.045	−1.356	0.529	−0.053	0.058	−0.917	0.796	0.107	0.054	1.982	0.2
Sham, Roff	Sham, Ron	0.076	0.045	1.676	0.34	0.102	0.058	1.77	0.293	0.019	0.054	0.356	0.984
Real, Ron	Sham, Ron	0.137	0.045	3.033	**0.015**	0.155	0.058	2.687	**0.04**	−0.088	0.054	−1.626	0.368

*Real, real nGVS condition; Sham, sham nGVS condition; Roff, without rubber condition; Ron, with rubber condition; COP-TL, total path length of movement of foot center of pressure; Vel-ML, velocity of the COP movement in the mediolateral direction; EMG-SOL, averaged amplitude of the electromyography of the soleus muscle; P-value adjusted for comparing a family of 4, bold letters indicate statistically significant results (p < 0.05)*.

Table 4 shows the results of the correlation analysis. There was no significant correlation in all conditions COP-related parameters and EMG-related parameters.

**Table 4:** Results from the Pearson's correlation.

		**Sham-nGVS**								**Real-nGVS**							
		**Without rubber**				**With rubber**				**Without rubber**				**With rubber**			
		**Eyes-open**		**Eyes-closed**		**Eyes-open**		**Eyes-closed**		**Eyes-open**		**Eyes-closed**		**Eyes-open**		**Eyes-closed**	
**Parameter 1**	**Parameter 2**	**r**	***p***	**r**	***p***	**r**	***p***	**r**	***p***	**r**	***p***	**r**	***p***	**r**	***p***	**r**	***p***
COP-TL	EMG-TA	0.029	0.912	−0.254	0.325	0.063	0.809	−0.155	0.553	0.069	0.791	0.122	0.642	−0.031	0.905	0.444	0.074
COP-TL	EMG-SOL	0.138	0.597	0.16	0.539	−0.095	0.718	0.196	0.45	0.026	0.921	−0.254	0.326	0.138	0.598	0.421	0.093
Vel-ML	EMG-TA	0.072	0.784	−0.378	0.134	−0.08	0.759	−0.408	0.104	−0.139	0.594	0.099	0.705	0.211	0.417	0.392	0.12
Vel-ML	EMG-SOL	0.027	0.919	0.136	0.604	−0.026	0.921	0.253	0.327	0.106	0.686	−0.252	0.33	0.18	0.489	0.417	0.096
Vel-AP	EMG-TA	−0.09	0.731	−0.094	0.719	0.197	0.449	0.049	0.852	0.093	0.721	0.087	0.74	−0.331	0.195	0.44	0.077
Vel-AP	EMG-SOL	0.206	0.428	0.088	0.738	−0.249	0.336	0.19	0.466	−0.171	0.513	−0.095	0.717	0.098	0.707	0.309	0.227
EMG-TA	EMG-SOL	0.027	0.917	−0.144	0.581	−0.045	0.862	−0.285	0.268	0.306	0.232	−0.102	0.696	0.309	0.227	−0.087	0.74

*COP-TL, total path length of movement of foot center of pressure; Vel-ML, velocity of the COP movement in the mediolateral direction; Vel-AP, velocity of the COP movement in the anteroposterior direction; EMG-TA, averaged amplitude of the electromyography of the tibial anterior muscle*.

## Discussion

This study aimed to investigate the effects of visual and somatosensory information on body sway and muscle activity during nGVS. We found a significant increase in COP-TL, Vel-ML, and EMG-SOL with rubber foam by nGVS (1 mA), and Vel-AP was increased by nGVS without eye and rubber effects. Rubber-foam significantly affected the EMG-SOL during nGVS. On the other hand, there were no effects on all parameters from visual information, and there was no significant correlation between the COP parameters and EMG activity parameters. These findings indicate that nGVS (1 mA) increases body sway and soleus muscle activity, especially on rubber-foam, without the effect of visual information in healthy young people.

There was a significant interaction effect of stimulation and rubber-foam on COP-TL and Vel-ML, and the *post-hoc* comparison revealed a significant increment of COP-TL and Vel-ML on rubber under real nGVS conditions. Further, there was a significant effect of stimulation on Vel-AP, and a *post-hoc* test revealed a significant increase in Vel-AP by nGVS unaffected by visual information and rubber-foam. These findings indicate that nGVS (1 mA) increases body sway partially depending on foot somatosensory condition and/or mechanically unstable foot plate, and not depending on visual condition. Rubber foam can make somatosensory input from feet unreliable and also generates mechanical consequences for balance control (Horak et al., 1990; Macedo et al., 2015), and vestibular contribution to postural control can be increased (Fujimoto et al., 2009). nGVS can modulate the threshold of the vestibular response (Kwan et al., 2019) against the signals from head movements and the excitability of the vestibulospinal response (Matsugi et al., 2020). Therefore, one possible mechanism of increment of body sway by nGVS (1 mA), if the stimulus intensity is in supratidal, is the increase in the threshold of the vestibulospinal response for postural control because the contaminating intensive noise stimulation generally disturbs signal detection (Fallon et al., 2004).

A previous study (Inukai et al., 2018b) reported that nGVS (1 mA) decreased body sway in young healthy people, as estimated by COP-TL, Vel-ML, and Vel-AP, and this intensity is the same as that in the current study. Therefore, we hypothesized that 1 mA-nGVS could decrease body sway in young healthy adults before starting the examination. However, another study reported that nGVS (intensity: 1 mA, duration: 30 s) was found to have no effect on postural stability in a healthy young population (approximately 23 years old) (Nooristani et al., 2019). Another previous study reported that the nGVS effect does not appear in a young healthy population with small postural sway on baseline measurement (Inukai et al., 2018b). Furthermore, Iwasaki and colleagues reported that low intensity nGVS (about 0.3 mA) decreases postural sway, but higher intensity (about 0.5 mA) increases postural sway in patients with vestibular dysfunction (Iwasaki et al., 2014; Sprenger et al., 2020). These findings indicate that nGVS effects depend on its intensity and the postural stability during pre-stimulation. In our study, an intensity of 1 mA might have been high, consequentially increasing postural sway, in other words, an intensity of 1 mA might be enough to disturb postural control in young adults.

Square wave pulse GVS changes the excitability of the spinal motor neuron pool in a polarity-dependent manner (Kennedy and Inglis, 2001) and typically induces body sway to the anodal side (Fitzpatrick and Day, 2004) accompanied by contraction of the lower limb muscle (Ali et al., 2003). This GVS-induced body sway increases depending on the difficulty of postural control, such as standing on unreliable foam and closing of eyes (Welgampola and Colebatch, 2001). In this study, before the examination, body sway to the anodal side was induced in all participants by square wave pulse GVS (intensity: 3 mA, duration: 200 ms), indicating that all participants responded to GVS-induced body sway. In this study, EMG activity in SOL was significantly increased in the rubber-foam condition, especially during nGVS. Based on these findings and the results, the increment of body sway by nGVS on rubber-foam may originate from muscle contraction induced by the vestibulospinal response. However, there was no evidence of causality and correlation of body sway and EMG activity because our results showed that there was no significant correlation between COP-related and EMG-related parameters. Dizziness or vertigo is one of the most frequent symptoms in patients with vestibular losses (Jeong et al., 2013). These symptoms originate from an increase in the variability of body sway, as well as from the patient's compensatory increase in foot muscle contractions (Schniepp et al., 2013). Therefore, it is possible that, in this study, muscle contraction increased to prevent falls, because GVS induces dizziness. Our results, along with those of previous studies, may not explain the increase in body sway induced by nGVS (1 mA) on rubber-foam. Future studies should investigate whether an increase in EMG activity caused body sway due to nGVS.

Visual information is often known to contribute not only to voluntary motion control (Yoshimura et al., 2010) but also postural control (Lim et al., 2019; Bonaventura et al., 2020) regardless of self-perception in body sway (Guerraz and Bronstein, 2008). On the other hand, the body sway induced by square wave pulse GVS with eyes closed is dramatically reduced when eyes are open (Fitzpatrick and Day, 2004). This phenomenon is explained by the compensation of visual information for the GVS-induced sensation of head movement. These findings support the hypothesis that nGVS effect increases in eyes closed condition. However, in the current study, there was no effect of eye condition on body sway and muscle activity. The finding that obstructing visual inputs might be unnecessary to obtain the effects of nGVS on body sway is consistent with those from a previous study that showed that nGVS affects body sway when participants' eyes are open (Inukai et al., 2018b). Therefore, the mechanism of increasing body sway with nGVS (1 mA) may be different from the mechanism of inducing body sway with square wave pulse GVS. Future studies should further investigate these mechanisms.

There are some methodological considerations for this study. The COP and EMG measurements were repeated under four conditions (eyes open/closed and with/without rubber), and further baseline and stimulation trials were conducted in one day. We made the order random and set a long measurement interval to reduce the effects of repeating measurements, but some effects might remain. Second, we did not control the amount of activity that participants were allowed to perform beforehand, such as sports or balance exercises, for more than a week between the sham and real nGVS conditions. Therefore, activity that occurred between the trials might have affected the results. Third, we considered two muscles: TA and SOL. The SOL is most typically tested muscle for postural control (Ivanenko and Gurfinkel, 2018) and for GVS effectiveness (Fitzpatrick and Day, 2004). The TA is an antagonist and collaborator of the soleus muscle (Di Giulio et al., 2009). The activity of the tibialis anterior muscle inhibits the soleus muscle, and sometimes they contract at the same time to increase the stiffness of the foot for postural control (Di Giulio et al., 2009). These muscles play a very important role in postural control; however, it is not possible to estimate overall postural control by observing these two muscles alone. Therefore, in future studies, we should investigate the effect of nGVS on other muscles for postural control, such as the quadriceps, hamstrings, gluteus medius, gluteus maximus, and erector spinae.

In conclusion, we investigated the effect of nGVS, visual information, and rubber-foam conditions on COP-TL, Vel-ML, Vel-AP, EMG-TA, and EMG-SOL. nGVS is often used to improve balance in patients with vestibular disorders, or in elderly people. Conversely, the current study suggested a possible decrease in balance in young healthy people with 1 mA-nGVS. There is no evidence of after-effects of 1 mA-nGVS on postural stability, but our results suggest that when nGVS is used for treatment in a young population, the stimulation intensity and use of rubber-foam should be considered. Furthermore, the manipulation of visual information may not be necessary.

## Data Availability Statement

The raw data supporting the conclusions of this article will be made available by the authors, without undue reservation.

## Ethics Statement

The studies involving human participants were reviewed and approved by the ethics committee of Shijonawate Gakuen University. The patients/participants provided their written informed consent to participate in this study.

## Author Contributions

AM initially designed this study. Experimental equipment was provided by KO and NM. The experiments were conducted by AM, KO, and NM. Data analyses were conducted by AM. AM initially wrote the manuscript, and all authors revised the manuscript. All authors contributed to the article and approved the submitted version.

## Conflict of Interest

The authors declare that the research was conducted in the absence of any commercial or financial relationships that could be construed as a potential conflict of interest.
